# Genome Sequence of *Bacillus subtilis* MB73/2, a Soil Isolate Inhibiting the Growth of Plant Pathogens *Dickeya* spp. and *Rhizoctonia solani*

**DOI:** 10.1128/genomeA.00238-13

**Published:** 2013-05-16

**Authors:** Dorota M. Krzyzanowska, Adam Iwanicki, Adam Ossowicki, Michał Obuchowski, Sylwia Jafra

**Affiliations:** Laboratory of Biological Plant Protection, Intercollegiate Faculty of Biotechnology UG&MUG, University of Gdańsk, Gdańsk, Polanda;; Laboratory of Molecular Bacteriology, Intercollegiate Faculty of Biotechnology UG&MUG, Medical University of Gdańsk, Gdańsk, Polandb

## Abstract

*Bacillus subilis* MB73/2 is a Gram-positive bacterium isolated in Poland from a meadow soil sample. When tested *in vitro*, the strain shows strong antagonism toward plant pathogens—the soft rot-causing bacteria *Dickeya* spp. and the crown rot fungus *Rhizoctonia solani*. Here, we present the genome sequence of MB73/2.

## GENOME ANNOUNCEMENT

*Bacillus subtilis* bacteria are soil-dwelling, Gram-positive organisms, often present in the rhizosphere. *B. subtilis* is considered a potent biological control agent due to the production of a variety of antibiotics, many of which inhibit the growth of plant pathogens. The antimicrobials produced by bacilli are predominantly ribosomal or nonribosomal peptides, exhibiting resistance to proteolytic hydrolysis, high temperature, and unfavorable pH (1). Low nutritional requirements and the formation of spores, which make the microorganisms easy to multiply and store, are other advantages of *B. subtilis* bacteria as potential biological control agents.

The *B. subtilis* strain MB73/2 is a meadow-soil isolate obtained in Zulawy, Poland. The strain shows strong *in vitro* antagonism toward plant pathogens responsible for significant losses in potato crops—the soft rot-causing bacteria *Dickeya* spp. (2) and the crown rot fungus *Rhizoctonia solani* (D. M. Krzyzanowska and A. Ossowicki, unpublished data). The mechanism underlying the antagonism toward both pathogens remains unknown. To facilitate our research on the identification of the responsible compound(s), we have performed genome sequencing of the MB73/2 strain.

Whole-genome shotgun sequencing of *B. subtilis* MB73/2 was performed at Baseclear (Leiden, The Netherlands) using the Illumina CASAVA pipeline version 1.8.2. The CLC Genomics Workbench version 5.5.1 (CLC bio, Aarhus, Denmark) was used to assemble the raw FASTQ sequences into 44 contigs of a total length of 4.17 Mb (645× coverage). The contigs were then assembled into 35 scaffolds using the SSPACE Premium scaffolder version 2.2 (3), and the gaps in the scaffolds were closed using the GapFiller version 1.11 (4).

The 35 scaffolds were concatenated into a single pseudomolecule. A spacer was introduced between the scaffolds, providing starts and stops in all six reading frames. Automatic annotation of the MB73/2 genome sequence was performed using the IGS Annotation Engine (5) (Institute for Genome Sciences, University of Maryland School of Medicine) (http://ae.igs.umaryland.edu/cgi/index.cgi). The Manatee tool was used for viewing the data (http://manatee.sourceforge.net).

The genome sequence of *B. subtilis* MB73/2 comprises 4,171,953 bp, with an average GC content of 43.4%. The annotation revealed 4,429 open reading frames (ORFs), 5 rRNA gene operons, and 76 tRNA genes. From the identified ORFs, 3,965 were assigned functions (89.5%). The genome was analyzed for the presence of genes encoding polyketide synthetases (PKS) and nonribosomal peptide synthetases (NRPS). One PKS coding cluster was revealed. As for the NRPS, a surfactin synthesis cluster was found; however, our biochemical study excluded the role of this lipopeptide in the growth inhibition of *Dickeya* spp. (M. Obuchowski, unpublished data). No genes involved in the synthesis of lipopeptides from the iturin family were present. MB73/2 harbors individual genes of the plipastatin (fengycin) synthesis pathway; however, not all building blocks of this plipastatin-like cluster are characteristic for the synthesis of plipastatin—genes encoding novel NRPS domains are also present. This may result in the production of a peptide with a different biological activity.

### Nucleotide sequence accession numbers.

This whole-genome shotgun project has been deposited at DDBJ/EMBL/GenBank under the accession number AOTY00000000. The version described in this paper is the first version, AOTY01000000.
